# A Practical Guide to Approaching Biased Agonism at G Protein Coupled Receptors

**DOI:** 10.3389/fnins.2017.00017

**Published:** 2017-01-24

**Authors:** Jaimee Gundry, Rachel Glenn, Priya Alagesan, Sudarshan Rajagopal

**Affiliations:** ^1^Trinity College of Arts and Sciences, Duke UniversityDurham, NC, USA; ^2^Department of Medicine and Biochemistry, Duke University Medical CenterDurham, NC, USA

**Keywords:** G protein coupled receptor, biased agonism, arrestins, G proteins, GRKs

## Abstract

Biased agonism, the ability of a receptor to differentially activate downstream signaling pathways depending on binding of a “biased” agonist compared to a “balanced” agonist, is a well-established paradigm for G protein-coupled receptor (GPCR) signaling. Biased agonists have the promise to act as smarter drugs by specifically targeting pathogenic or therapeutic signaling pathways while avoiding others that could lead to side effects. A number of biased agonists targeting a wide array of GPCRs have been described, primarily based on their signaling in pharmacological assays. However, with the promise of biased agonists as novel therapeutics, comes the peril of not fully characterizing and understanding the activities of these compounds. Indeed, it is likely that some of the compounds that have been described as biased, may not be if quantitative approaches for bias assessment are used. Moreover, cell specific effects can result in “system bias” that cannot be accounted by current approaches for quantifying ligand bias. Other confounding includes kinetic effects which can alter apparent bias and differential propagation of biological signal that results in different levels of amplification of reporters downstream of the same effector. Moreover, the effects of biased agonists frequently cannot be predicted from their pharmacological profiles, and must be tested in the *vivo* physiological context. Thus, the development of biased agonists as drugs requires a detailed pharmacological characterization, involving both qualitative and quantitative approaches, and a detailed physiological characterization. With this understanding, we stand on the edge of a new era of smarter drugs that target GPCRs.

## Introduction

G protein-coupled receptors (GPCRs) are the most common receptors in the genome and one of the largest drug targets for neuroendocrine disease (Overington et al., 2006). Classically, drugs targeting these receptors have been considered along the spectrum from antagonists to partial agonists to full agonists, which block, partially activate or fully activate, respectively, all of the signaling pathways downstream of a receptor. Over the past two decades, we have now appreciated a different phenomenon, biased agonism (in contrast to “balanced agonism”), the ability of some ligands to selectively activate some signaling pathways while blocking others (Rajagopal et al., 2010). Biased agonism was first noted as a reversal of the order of potencies for different ligands between alternative G protein signaling pathways (Kenakin, 1995). While the study of biased agonism has largely focused on GPCRs, it is likely to occur in other receptor types as well (Zheng et al., 2012). A biased response is due to a combination of two distinct phenomena, ligand bias and system bias (Kenakin and Christopoulos, 2013b). Ligand bias, or “true” biased agonism, refers to differences in signaling due to the molecular variation that governs the interaction between the ligand and the transduction proteins at the receptor. Ligand bias is thought to be due to the stabilization of distinct receptor conformational states that differentially activate these alternative signaling pathways (Kahsai et al., 2011; Liu et al., 2012; Wacker et al., 2013). For GPCRs, the easiest bias to observe is that between selective activation of heterotrimeric G proteins (G protein-bias) and β-arrestin (β-arrestin-bias) adapter proteins (Wei et al., 2003). This is because G proteins and β-arrestins typically activate distinct signaling pathways, with G proteins typically activating second messengers and β-arrestins regulating receptor desensitization, internalization and activation of MAP kinases (DeWire et al., 2007). In contrast, system bias, or “apparent” biased agonism, is a reflection of the differences in measurements of biochemical amplification at the tissue, cellular, or *in vitro* level between the assays that are being used (Onaran and Costa, 2012). Thus, system bias has contributions from true differential amplification of signaling pathways (amplification bias) and the assays used to assess these signaling pathways (observation bias). In the development of biased agonists, it is critical to apply approaches that can separate ligand bias, which should be present across different assays, from system bias.

Biased agonists are expected to have different functional and physiological consequences from conventional balanced agonists, given that they activate only a select portion of a receptor's signaling cascade while inhibiting others (Whalen et al., 2011). Because so many drugs target GPCRs, biased agonism holds the promise of developing a whole new class of “smarter” drugs that selectively target therapeutically relevant signaling pathway with fewer side effects from non-selective activation or blockade of other signaling pathways. A few therapeutics in the clinic have since been shown to act as biased agonists, which may explain why some drugs have greater efficacy than others within the same class (Kim et al., 2008). Conversely, failure to account for the potential of biased agonism may lead to the development of pharmaceuticals that may target the relevant signaling pathway while, at the same time, activating pathways leading to intolerable side effects. The goal of this perspective is to highlight examples of drug development of biased agonists, current limitations in their characterization and a general approach to characterizing the pharmacology of this promising new class of drugs.

## The promise of biased agonism

For biased agonists to be developed as drugs, a clear understanding of their physiological effects must be determined. Biased agonists targeting a number of disease states have been and are currently being developed (reviewed in Whalen et al., 2011; Kenakin and Christopoulos, 2013b), and a review of all of those studies is beyond the scope of this perspective. Rather, we will focus on biased drug development at two receptors that are important in the nervous system: The dopamine D2 receptor and the μ-opioid receptor (μOR). Dopamine D_2_ receptors were originally thought to affect schizophrenia through Gα_i_/Gα_0_-mediated inhibition of adenylyl cyclase (Girault and Greengard, 2004). Based on that understanding, one would expect that blockade of G protein-mediated D2 signaling would be sufficient to treat schizophrenia. However, behavioral and biochemical evidence has since shown a central role of β-arrestin 2 in signal transduction by D_2_ dopamine receptors through the regulation of the AKT-GSK3 pathway (Beaulieu et al., 2007), through the formation of a protein complex composed of β-arrestin 2, AKT, and PP2A that promotes the dephosphorylation of AKT in response to dopamine. Lithium, a common drug used to treat bipolar disorder and other psychiatric illnesses, targets this protein complex, as do a wide array of antipsychotic medications (Masri et al., 2008). In β-arrestin 2 knockout mice, the behavioral effects of lithium treatment are lost, and the mice display defects in behaviors known to be regulated by dopamine (Beaulieu et al., 2008). More recently, a β-arrestin-biased D2 receptor agonist has been developed (Allen et al., 2011) that has distinct effects from balanced agonists in a mouse model of schizophrenia (Park et al., 2016).

The μOR is the target for endogenous enkephalin peptides and exogenous opioid analgesics including morphine, which act as agonists. Enkephalins are balanced agonists for G protein- and β-arrestin-mediated pathways, whereas morphine is biased toward G protein-mediated signaling, with a considerable reduction of receptor phosphorylation and internalization (Bohn et al., 2004). However, β-arrestin 2 knockout mice have demonstrated amplified and prolonged morphine-induced analgesia compared to wild type mice, consistent with the presence of morphine-induced β-arrestin-mediated desensitization (Bohn et al., 1999). Furthermore, β-arrestin 2 knockout mice are protected from the side effects of morphine such as respiratory depression and constipation, which suggests that β-arrestin-mediated pathways control these peripheral side effects (Bohn et al., 2000). Recently, G protein-biased μOR agonists have been developed using different strategies (DeWire et al., 2013; Manglik et al., 2016). These drugs provide analgesia in animal models without the side effects of respiratory depression and tolerance (DeWire et al., 2013; Manglik et al., 2016), and one of these compounds has already shown promise in early phase clinical trials in humans (Soergel et al., 2014).

## Limitations to identifying biased agonists

While there is considerable promise in the development of biased agonists as therapeutics, there are a number of considerations that must be addressed when characterizing a biased agonist, from the pharmacological to the physiological levels (Table 1).

**Table 1:** **Limitations to the assessment of biased agonism and approaches to minimize them**.

**Problem**	**Solution**
Ensure that the ligand is biased	Choose assays to minimize difference in amplification Use qualitative and quantitative approaches for assessing ligand bias and removing effects of system bias
Confounding by cell-specific effects	Use cells that are as close to physiological as possible Validate findings from heterologous system in more physiologically relevant cell type
Unexpected propagation of bias	Obtain data from multiple time points to ensure that bias persists over biologically relevant time scale Assess different reporters downstream of the same effector to ensure similar degrees of bias
Complex/Unexpected physiology	Test effects of biased agonists in physiologically relevant cell types and animal models of disease

### Make sure your ligand is actually biased

Many older studies assumed that a ligand was biased compared to a balanced agonist if there was a significant difference in efficacies or potencies through different signaling pathways. However, large differences in potency and efficacy can be due to system bias and not ligand bias (Onaran and Costa, 2012). One of the first methods for properly identifying biased ligands was by identifying a change in the rank order of potency of ligands (Kenakin, 1995). Over the past few years, a number of approaches have been developed to identify and quantify ligand bias through the calculation of “bias factors” (reviewed in Kenakin and Christopoulos, 2013a). While a full discussion of the details of these different approaches is beyond the scope of this perspective, we discuss some of their advantages and disadvantages below (see *General Approach*).

### Avoid confounding by cell-specific effects

Even with our current approaches for assessing bias, it is still possible that the effects of system bias cannot be fully accounted for. For example, the bias factor approaches based on the operational model are best suited for cases in which the major difference is a change in receptor number or immediate downstream amplification, as the τ factor (an estimate of efficacy) is equal to receptor concentration divided by a constant for system amplification (Black and Leff, 1983). The operational model cannot correct for examples in which other cofactors that affect signaling, such as GRKs, are differentially expressed. For example, GRK2 overexpression is known to phosphorylate the μOR and increase β-arrestin recruitment to the receptor in response to morphine (Zhang et al., 1998). However, a recent study has shown that GRK2 activity at the μOR generates a unique conformation of the receptor that is associated with differential activity (Nickolls et al., 2013). This type of behavior cannot be accounted for using pharmacological methods for quantifying bias.

### Watch for unexpected propagation of bias

A recent study by Klein Herenbrink et al. (2016) highlighted that apparent bias may change depending on the time and pathway assessed. At the D2 dopamine receptor, they found that there was a significant effect of ligand-binding kinetics and the temporal pattern of receptor-signaling processes on the observed bias of different ligands. These differences even led to some examples of reversals in the direction of bias. Most methods for determining bias factors assume equilibrium conditions, a situation which is clearly absent when there is a significant kinetic effect. Also, the authors found that different reporters of the same pathway could have different degrees of amplification and estimated bias. At the μOR, a robust correlation was found between off-rate kinetics for ligands and slower receptor dephosphorylation and β-arrestin dissocation (Sianati, 2014), suggesting similar behaviors at other GPCRs. These kinetic effects must be considered in the assessment of bias.

### Characterize the physiological effects of the biased agonist

It is common for the pharmacological effects of a drug to not correspond with its *in vivo* activity, due to off-target effects or unexpected biology. This is especially true for biased agonists, which have more complex effects than simple agonists or antagonists. For example, SII angiotensin is a synthetically modified form of angiotensin II that binds the angiotensin type 1A receptor (AT_1A_R) (Holloway et al., 2002). SII is unable to activate Gα_q_ signaling but retains the ability to recruit β-arrestin 2, which would be expected to result a loss of calcium signaling with increased desensitization (Wei et al., 2004). However, SII was found to act as a calcium sensitizer in cardiomyocytes (Rajagopal et al., 2006; Monasky et al., 2013) through a novel β-arrestin regulatory mechanism. Subsequent work, however, has shown that the signaling pattern induced by SII is much more complex, and involves activation of other G protein-dependent effects, suggesting that the relationship between observed bias and physiological effects is more complex (Sauliere et al., 2012). Thus, sometimes it can be difficult to establish a clear connectivity between biased coupling and cellular behavior. For example, at the urotensin receptor, ligands which differentially activated G_q_, G_13_, G_i/o,_and β-arrestin, do not display clear patterns for their effects on cell death, migration and adhesion (Brule et al., 2014). It is critical to characterize signaling pathways activated by biased agonists in physiologically relevant tissues, as these can be very different from heterologously expressed cells.

## A general approach to identifying and characterizing biased agonists

**Figure 1:**
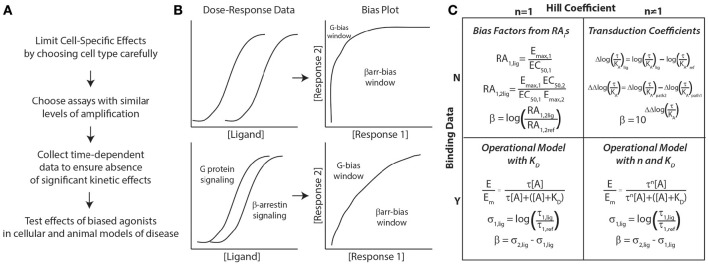
**General approach to assessing biased agonism. (A)** Considerations for assay development in characterizing biased agonists. **(B)** Bias plots are generated by converting dose-response data for 2 signaling pathways (G protein and β-arrestin signaling here) to response 1 vs. response 2 data (here β-arrestin vs. G protein signaling). If there is significant amplification between assays, the window for identifying G protein-biased ligands decreases significantly (top panel). To identify both G protein- and β-arrestin-biased, assays with similar levels of amplification should be used (bottom panel). **(C)** Approaches to quantifying bias based on the presence of binding data (dissociation constant, K_D_) and whether the concentration-response data is best fit with a Hill coefficient (*n*) of non-unity. All of these approaches can yield a bias factor, β. For more details on these different approaches, please refer to the text.

Based on these considerations, we recommend the following approach to identify biased agonists (Figure 1A). First, to limit possible cell-specific effects, cells that are as close to physiologically relevant as possible should be used for the assays used to test bias. This can be difficult, however, as most physiologically relevant cell lines are difficult to transfect and not suited to most pharmacological assays. Therefore, it is important to confirm, after a potential biased agonist has been identified, that its biochemical effects are observed in a physiological relevant cell type. Second, in choosing the assays for different signaling pathways, they should have similar levels of amplification, i.e., these assays should generate similar signals for the same concentration of ligand (Rajagopal et al., 2010). This provides a larger window for identifying biased agonists (Figure 1B). For example, assays that measure second messengers downstream of G proteins, such as cyclic AMP (cAMP) or calcium, have significant amplification. This is in contrast to recruitment assays of G proteins or β-arrestins to the receptor using bioluminescence resonance energy transfer (BRET), in which the spatial proximity of a luciferase (RLuc) -tagged receptor to a yellow fluorescent protein (YFP)-tagged effector results in energy transfer. In a BRET assay, the YFP:Rluc ratio indicates the degree of recruitment, with virtually no amplification. Assays that report on receptor internalization can be useful in determining receptor distribution in response to ligand stimulation, as shortly after β-arrestin recruitment, receptors undergo endocytosis and rapid or slow recycling. Using reporters that are significantly distal to the receptor runs the risk that they may report on other effectors, e.g., MAP kinase activation is regulated both by G proteins and β-arrestins. Third, to avoid confounding from potential kinetic effects, it is important to collect time-dependent data to ensure that any bias persists across a valid biological time scale. Lastly, the effects of biased agonists should be tested in cellular and animal models, as little may be known about the physiological effects of a biased agonist.

With respect to the specific methods used to quantify ligand bias, both qualitative and quantitative methods should be used to identify potentially biased ligands (Rajagopal et al., 2011). Most quantative approaches for bias result in the calculation of a “bias factor” that quantifies the degree of ligand bias numerically. The details of bias factor calculations are beyond the scope of this perspective, and the interested reader should refer to the specific citations below. First, use “bias plots” to qualitatively identify potentially biased ligands (Figure 1B) (Gregory et al., 2010). If a ligand does not demonstrate bias on the bias plot (has a similar response-response curve on the bias plot to the balanced agonist) but does have a significant bias factor, it is likely that the bias factor calculation is in error. This is because errors in a bias factor can be introduced at multiple stages in the fitting of concentration-response data depending on the technique used. If the data is fit well with a simple dose-response equation with a Hill coefficient of 1, the most straightforward approach to calculate a bias factor is by the logarithm of ratios of relative intrinsic activities (Griffin et al., 2007; Rajagopal et al., 2011) (Figure 1C). This calculation does not require additional information on ligand binding nor a complex fitting routine (it just requires E_max_s and EC_50_s for the different assays) that could introduce errors into the bias factor. An alternative approach is to calculate transduction coefficients (Kenakin et al., 2012), although that should be mathematically identical with bias factors obtained from intrinsic relative activities when the Hill coefficient is 1 (Griffin et al., 2007).

If binding data for ligands and a reference agonist are available, fitting to an operational model (Black and Leff, 1983) can yield both bias factors and estimates of efficacy. This estimate of efficacy (the effective signaling, σ) (Rajagopal et al., 2011), is closely related to intrinsic efficacy, ε, from classic pharmacological theory (Onaran et al., 2014). The advantage of this estimate of efficacy is that it provides information to the degree of agonism of the ligand tested, e.g., whether the ligand is a weak partial agonist or a full agonist. This data is not provided by a bias factor, which only gives an estimate of the relative efficacies of two signaling pathways compared to one another for a single ligand. As an example, a bias factor cannot differentiate between a weak partial agonist that is biased and a similarly biased full agonist; comparing their effective signaling can differentiate between such drugs. This approach should provide efficacy estimates even if the Hill coefficient is not unity.

If binding data is not unavailable and the Hill coefficient is not one, then the best approach to use is the calculation of transduction coefficients (Kenakin et al., 2012). In this approach, transduction coefficients [log(τ/K_A_)] are fit to the data along with an “apparent” dissociation constant; bias factors can be calculated from these transduction coefficients. For a partial agonist, in which the E_max_ for the ligand does not approach the maximal effect of the system, the EC_50_ approaches the dissociation constant for the ligand, K_D_. In that situation, the data will be well fit with the transduction coefficient equation. However, for full agonists, where E_max_ approaches the maximal effect of the system, there may not be a clear relationship between EC_50_ and K_D_. This can result in an ambiguous fit associated with relatively larger errors for estimates in transduction coefficients and bias factors.

## Conclusions

Drug discovery of biased agonists is an active area of research which has exploded over the past 5 years. In the development of biased agonists, it is critical that potential limitations in their characterization should be minimized. This means that we must confirm that the ligand is actually biased using qualitative and quantitative approaches, that there is no significant confounding from cell-specific effects, that there is not unexpected propagation or kinetic effects in signaling and that we understand the physiological effects of the biased agonists in cellular and animal models of disease. Using this general approach, a broad understanding of signaling by biased agonists from the pharmacological to the physiological level can be obtained and we can move forward in the development of these promising agents as novel therapeutics.

## Author contributions

All authors listed, have made substantial, direct and intellectual contribution to the work, and approved it for publication.

## Funding

SR is funded by NIH HL114643 and a Burroughs Welcome Career Award for Medical Scientists.

### Conflict of interest statement

The authors declare that the research was conducted in the absence of any commercial or financial relationships that could be construed as a potential conflict of interest. The reviewer HC and handling Editor declared their shared affiliation, and the handling Editor states that the process nevertheless met the standards of a fair and objective review.
